# Porosity and cement controlling the response of artificially cemented tailings under hydrostatic loading to high pressures

**DOI:** 10.1038/s41598-024-80937-7

**Published:** 2024-11-26

**Authors:** Nilo Cesar Consoli, João Vítor de Azambuja Carvalho, Alexia Cindy Wagner, Inácio Carvalho, João Paulo de Sousa Silva

**Affiliations:** 1 Graduate Program in Civil Engineering, Universidade Federal do Rio Grande do Sul, Porto Alegre, 90035-190 RS Brazil; 2 LGV (Laboratório de Geotecnia VALE), VALE S.A., Santa Luzia, 33040-900 MG Brazil

**Keywords:** Dry stacking, Iron ore tailings, Compaction, Portland cement, High pressures, Yielding, Civil engineering, Geology

## Abstract

Cemented tailings find various applications in mining, such as open-pit and underground backfill, dam decommissioning and filtered tailings stacking. This research investigates the compression behaviour of iron ore tailings (IOT) mixed with distinct amounts of Portland cement and compacted in different conditions through isotropic compression, pulse velocity, and unconfined compression tests. The results show the adequacy of the porosity/cement index (*η/C*_*iv*_) in predicting elastic and plastic characteristics of compacted filtered IOT - Portland cement blends, an original correlation that has not been reported by previous work. This index is useful in selecting the cement content and target density for essential parameters required to design cemented IOT stacks. Besides, both cement addition and compaction have promoted the tailings isotropisation (conversion of an anisotropic system to an isotropic one). The evolution of the Post-Yield Compression Line (PYCL) with cementation is shown. Finally, it is demonstrated that distinct initial (after compaction) porosities of uncemented specimens reach a unique PYCL after isotropic pressures above 100 MPa, and cemented specimens do not reach a unique PYCL even at 120 MPa of isotropic pressures. The results underscore the requirement of rigorous compaction control in the field and offer a methodology for the dosage and technological control of artificially cemented tailings.

## Introduction

Unlike natural soils, tailings are recently deposited materials formed by industrial processes and disposed of in different types of Tailings Storage Facilities. As a reason, the structure of tailings is highly dependent on the choice of deposition method. Here, structure is considered as the combination of fabric (originated from particle arrangement) and bonding (cohesive interactions between particles), as defined by Mitchell and Soga^1^. Regarding fabric, the slurry-deposited tailings in large impoundments are formed under high void ratios and continue to deform for long periods under self-weight solicitations^2,3^. Simulating this complex in-situ fabric is still the focus of debate and proposition of new reconstitution methods for laboratory specimens since retrieving undisturbed samples can be difficult due to the cohesionless nature of tailings^4–6^. Otherwise, when disposed of in dry stacks facilities, the tailings’ fabric is highly dependent on the compaction conditions (e.g., energy employed, moisture content at compaction). In these cases, independent of the deposition method, the tailings’ structure depends only on the fabric formed at the field.

Also, bonding can arise in tailings due to natural light cementation in hydraulic-disposed tailings^3,7^ or due to the addition of cementing agents in dry-disposed tailings. The latter situation has been studied to improve the stability of tailings in dry stacks^8,9^. The use of cementing agents and compaction gives rise to another structure in terms of bonding (in addition to fabric).

Usually, research on the role of cementation is limited to lower pressures, in which the bonds between particles provided by the cementing agent are still acting. However, cement may influence the compression behaviour of geomaterials up to high pressures^10^. One aspect of behaviour affected by cement is the location of the Post-Yield Compression Line (PYCL)^11–13^. The compression behaviour of cemented materials usually presents two sections (before and after the cementation break). The point at which the behaviour changes from cement-dominated behaviour to friction-dominated behaviour is termed yielding point. Thus, the PYCL refers to the steep linear portion after the yielding point, where cementation no longer controls the response. However, after bonds breakage (yielding), the cement can continue to influence the material’s behaviour and tends to change the PYCL location^11^. Thus, the effects of changing the PYCL location in the state space must be accounted for in the geomaterial’s constitutive description. A framework that captures the impact of the initial density, the mean effective stress, and the outcome of cement addition, describing different states in which such geomaterial can exist, is needed.

Besides, the behaviour of geomaterials has been extensively studied up to confinement levels of 1 MPa. Nevertheless, some cases involve higher-order pressures^14–17^. In underground mining, the high-stress conditions are associated with the backfilling of the mines with waste rock and reprocessed tailings^18^. In the open-pit mining industry, high-pressure problems may arise from the high dry stacks facilities due to the large quantities of tailings produced. Therefore, it is necessary to understand the behaviour of tailings for higher pressures than considered in conventional geotechnical testing practice.

Thus, in spite of the studies of uncemented soils over a broad range of pressures and densities^19–23^ and investigating the response of cemented sands thoroughly^11,24–27^, there is a paucity of research on the behaviour of artificially cemented tailings over a broad range of cement contents and stress levels. One of the few examples that can be found is the work of Jafari et al.^18^, who investigated the behaviour of tailings for cemented tailings backfill up to 6.5 MPa in confinement.

This paper aims to describe iron ore tailings–Portland cement compression behaviour on a spectrum of pressures, compacted porosities, and cement contents broader than previously reported. For the first time, the compression behaviour of cemented tailings is investigated at high pressures and related to the porosity/cement index framework. For this, isotropic compression tests under confining pressures up to 120 MPa, unconfined compression strength (UCS) tests, and ultrasonic pulse velocity (UPV) tests were conducted, considering distinct amounts of Portland cement under loose to dense compaction conditions. The elastic and plastic characteristics of different mixtures were examined. The porosity/cement index was applied to assess the pre-yield behaviour, while the cement content was used to describe the post-yield behaviour. The isotropisation of the compacted mixes was also evaluated. Furthermore, the influence of distinct initial (after compaction) porosities and cement contents on the PYCL location was investigated.

## Materials and methods

The experimental program comprises unconfined compression and ultrasonic pulse velocity tests conducted on iron tailings–cement blends over a broad range of cement contents and porosities. Also, isotropic compression tests up to 120 MPa were carried out on the mixtures to evaluate the influence of dry density and cement content on the mean effective pressure–specific volume, strains isotropy, yielding, and initial bulk modulus.

### Materials

**Fig. 1:**
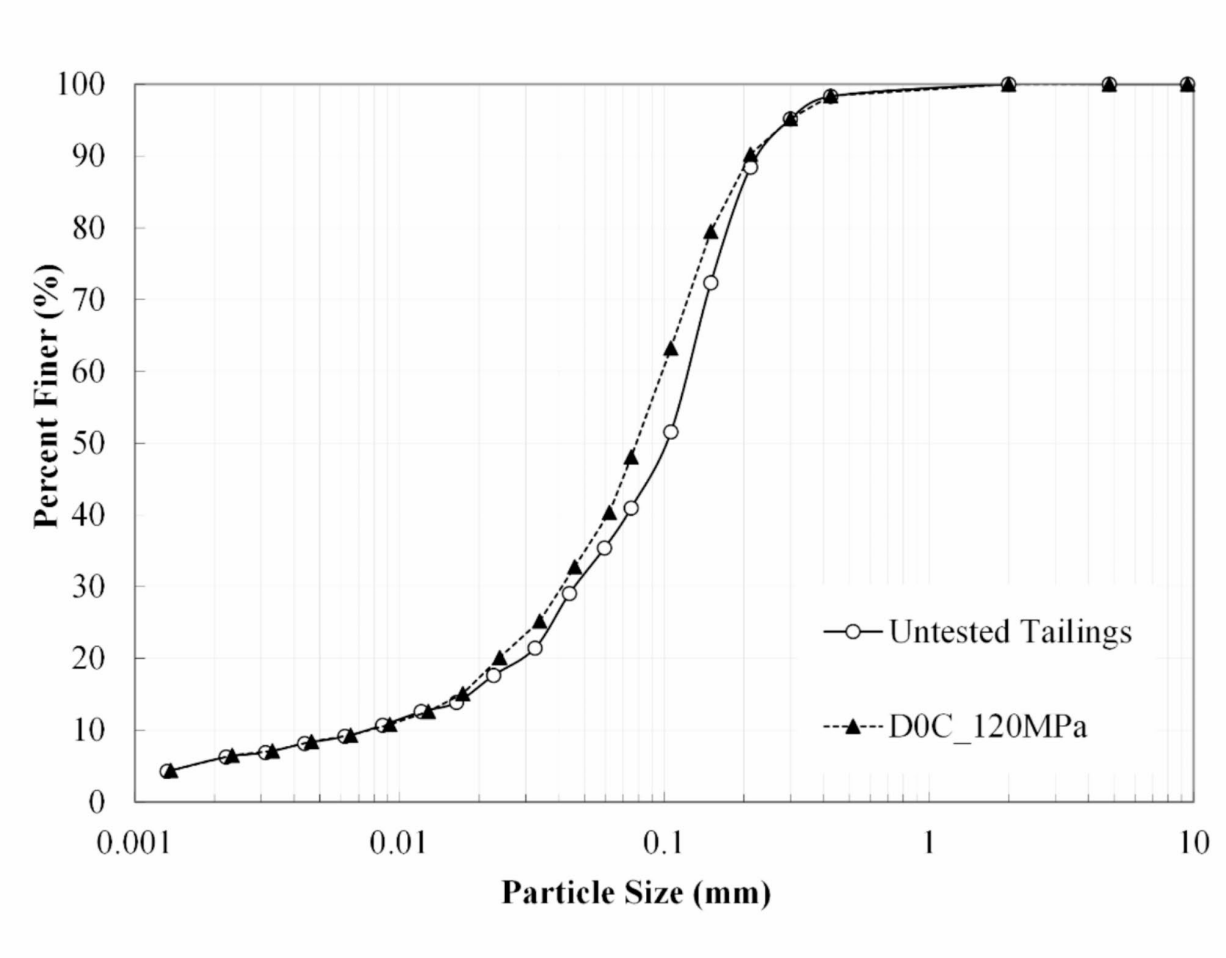
Particle size distribution of untested tailings and uncemented dense tailings after isotropic compression up to 120 MPa.

The iron ore tailings (IOT) studied are from a mine in the Quadrilátero Ferrífero region in Minas Gerais, southeast of Brazil. The tailings were obtained after the filtering process at the exit of the beneficiation plant. The particle size distribution^28^ for the untested tailings (Fig. 1) indicates 1.7% medium sand, 57.4% fine sand, 34.7% silt, and 6.3% clay size, and coefficients of curvature (*C*_*c*_) and uniformity (*C*_*u*_) were 2.0 and 17.3, respectively. The Atterberg limits (non-plastic tailings) and the specific gravity (*G*_*s*_ = 2.92) were evaluated, respectively, according to ASTM D4318^29^ and ASTM D854^30^. The compaction characteristics were assessed using the standard Proctor compaction effort (*w*_*opt*_ = 11.6% and *g*_*d max*_ = 19.2 kN/m^3^) in agreement with ASTM D698^31^. Following the Unified Soil Classification System^32^ the material is classified as silty sand (SM). The IOT mineralogy was assessed by X-ray Diffraction (DRX). The tailings are mainly constituted of quartz (69.7%), iron oxide (24.0%), and aluminium oxide (4.8%), amongst others.

The cement used in this study was the high early strength (Type III) Portland cement (*G*_*S, PC*_ = 3.15), because of its fast strength gain, aiming to achieve significant strength during short curing periods.

### Methods

#### Moulding

The different tests were performed with tamped cylindrical specimens (50 mm in diameter and 100 mm in height). The uncemented tailings specimens were prepared by tamping the dry tailings in layers directly into a membrane attached to the bottom pedestal of the triaxial cell while those cemented were moulded following the undercompaction method^33^. A target dry unit weight (*γ*_*d*_) was defined for each specimen, and the equivalent iron tailings and Portland cement masses were calculated based on the specific dosage. Following Consoli et al.^34^, the porosity (*η*) is a function of the dry unit weight (*γ*_*d*_) of the mixture and Portland cement content (PC) (Eq. 1). Therefore, the unit weight of solids of each component (*γs*_*IOT*_, for IOT, and *γs*_*PC*_, for Portland cement) was considered for the porosity determination.

1 $$\eta =100 - 100\left\{ {\left[ {\frac{{{\gamma _d}}}{{1+\frac{{PC}}{{100}}}}} \right]\left[ {\frac{1}{{\gamma {s_{IOT}}}}+\frac{{\frac{{PC}}{{100}}}}{{\gamma {s_{PC}}}}} \right]} \right\}$$

The materials (i.e., IOT and cement) were mixed by hand at dry conditions until a homogeneous mixture was obtained. Afterwards, distilled water was added to achieve the optimum moisture content determined from compaction tests. The specimen was compacted into three layers, and the top of the intermediate layers was slightly scarified to guarantee adherence between them. Following, the specimen was measured, weighed, sealed, and cured in a humid room at 23 ± 2 °C for 28 days. Before being taken to testing, all the specimens were oven-dried for 24 h at 105 °C to guarantee the zero-moisture content condition and avoid suction effects. The intention was to maintain the same specimen condition for all performed tests because the program of isotropic compression tests required dry specimens.

#### Program of unconfined compression tests

Two target dry unit weights (17 kN/m^3^ and 19 kN/m^3^) and seven amounts of Portland cement contents (1%, 2%, 3%, 4%, 5%, 10% and 20%) were investigated in the program of UCS tests. The dry unit weights were selected to represent different compaction conditions of the tailings at the field (approximately 100% and 80% of the compaction degree considering the standard effort). The cement range selected aims to cover usual (up to 5%) and excessive (20%) amounts of Portland cement, considering the cost. The tests were performed using an automatic loading ram equipped with an electronic load cell (50 kN of system maximum capacity) at a constant displacement rate (1.14 mm/min) as per ASTM D2166^35^. Three specimens were tested for each dosage.

#### Program of ultrasonic pulse velocity tests

The UPV tests can determine artificially cemented tailings’ initial Shear Modulus (*G*_*0*_). The *G*_*0*_ value can be determined by the product between the bulk density and the square of the velocity of a shear wave passing through a homogeneous and elastic medium^1^. Therefore, UPV tests are non-destructive, which allows them to be performed on the specimens before the unconfined compression tests. To perform the tests, a special gel was used to couple transducers on the specimen’s top and bottom, and a device was used to emit shear waves (250 kHz) across the specimen while the propagation time was measured. This procedure permits determining the shear modulus at very small deformations (*G*_*0*_).

#### Program of isotropic compression tests

A series of isotropic compression tests were carried out on cemented compacted filtered IOT to evaluate the materials’ compression response. The characteristics of the specimens tested under isotropic compression are summarised in Table 1. Distinct dry unit weights (17 kN/m^3^ and 19 kN/m^3^) and four cement dosages (0%, 3%, 5%, and 20%) were selected to analyse the influence of compaction and cementation degree on the isotropic compression response. The dry unit weights were chosen as two possibilities for compaction in the field and allowed for convergence determination once different initial void ratios were obtained for each dosage. The cement contents selected were due to technical feasibility in the field. The 3% and 5% dosages represent small amounts of binder addition and are empirical limits for the solution’s economic viability. The dosage of 20% cement was selected as a boundary limit to study the full range of behaviour for IOT-Portland cement mixtures. These cement dosages are consistent with the current practice in cement-stabilized tailings for various applications, from dry stacking to backfill^8,18,36–38^. Furthermore, this experimental program provides six points (disregarding the two uncemented specimens) when analysed under the porosity/cement content index framework^25^. The number of specimens tested in this phase was limited in quantity because of operational difficulties and material restrictions since the equipment used in compression tests up to 120 MPa requires a large operation and mobilisation, and the samples cannot be reused. However, these few tests are essential to provide significant insights into the compression behaviour of cemented tailings.

**Table 1:** Main characteristics of the specimens submitted to isotropic compression tests.

Sample	Dry unit weight—*γ*_*d*_	Cement content—*C*	Porosity—*η*	Volumetric cement content—*C*_*iv*_	Porosity/cement content—*η/C*_*iv*_	Yield point—YP (kPa)	Initial bulk modulus—*B* (MPa)
	(kN/m^3^)	(%)	(%)	(%)			
L0C	17	0	40.26	–	–	300	129
L3C		3	40.64	1.66	24.52	590	287
L5C		5	41.41	2.72	15.25	4,290	739
L20C		20	42.44	10.85	3.91	23,160	2,973
D0C	19	0	33.48	–	–	400	145
D3C		3	33.82	1.85	18.31	605	420
D5C		5	34.75	3.03	11.47	4,390	1,235
D20C		20	35.52	12.12	2.93	39,890	4,741

The high-pressure triaxial isotropic compression tests consisted of incrementing the chamber pressure at a constant rate (1.2 MPa/minute) up to the desired 120 MPa value. This rate was considered appropriate as the strain rate has not been found to influence the results of isotropic compression to high pressures for sands^16^. The 120 MPa value was selected because it is the maximum pressure of the apparatus used, aiming to evaluate the IOT-Portland cement mixtures’ behaviour under extreme conditions. The use of an MTS in-vessel extensometer kit allowed the assessment of the volumetric strains during the entire isotropic compression tests on dry specimens. These instruments are designed for measuring axial and circumferential strains on a cylindrical specimen loaded in a high-pressure vessel (up to 140 MPa). Two axial extensometers (+ 5/− 2.5 mm maximum travel) fixed in opposed positions were used to assess the axial displacements. In turn, the circumferential strain was calculated by a chain system, which measures the circumference change deformation during the test (± 3.75 mm maximum chordal travel). The chain was positioned in the middle of the specimen, between the extremities of axial extensometers.

## Results and discussion

### Particle breakage

The particle size distribution (PSD) was evaluated with untested tailings and a sample retrieved after isotropic compression testing, considering an uncemented specimen with a dry unit weight of 19kN/m³. The PSD curves are shown in Fig. 1 and are very similar, indicating a low particle breakage due to isotropic compression. An adaptation of the relative breakage parameter—*B*_*r*_^39^ was employed to quantitatively assess the amount of breakage^20^. Hardin^39^ limited the lower limit for grain size to 0.074 mm for the index calculation. However, in this case, an upward shift in the PSD curve for this size range is observed in Fig. 1, indicating the creation of more particles smaller than 0.074 mm after testing. So, all the grain sizes of the distribution curves were used in the calculation. The *B*_*r*_ value obtained was 0.106 and was very low for the sample tested. This indicates that grain crushing is not a primary mechanism in the tailings’ isotropic compression up to very high stresses. Therefore, the influence of breakage was not included in the analyses performed.

### Tailings isotropisation by cement addition

The compaction process occurs in one main direction (axial), creating a cross-anisotropic fabric that influences the material’s response to the loadings. However, the hypothesis of isotropic behaviour reduces the number of variables in equilibrium equations, thus simplifying the solution and reducing the computational cost. As a reason, usual geotechnical engineering methods used in practice for predicting stresses and strains assume isotropy. Therefore, isotropisation makes the tailings behaviour more predictable with existing methods, as modelling anisotropic materials is still difficult.

**Fig. 2:**
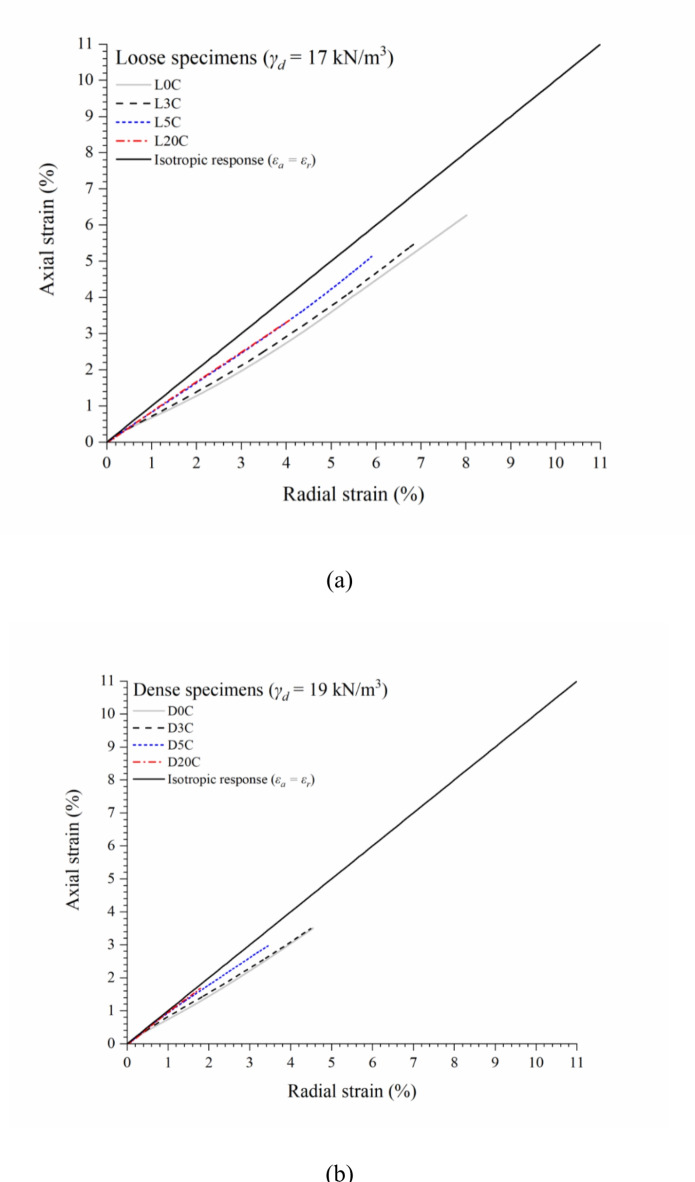
Relation between axial and radial strains during isotropic compression for (a) loose iron ore tailings-Portland cement mixtures and (b) dense IOT-Portland cement mixtures.

Figure 2a and b present the relations between axial and radial strains during isotropic compression tests performed on (a) loose and (b) dense specimens, respectively, considering cement contents of zero, 3%, 5%, and 20%. Table 1 summarises the specimens’ characteristics and test results. The tests are identified following the general nomenclature XyC, where X has to do with compaction conditions (L for loose and D for dense), y is the cement content, and C means Portland cement. From Fig. 2a and b, it is possible to observe that the addition of cement approximates the material’s compression response to an isotropic behaviour. However, there appears to be a stabilisation after 5% cement, where further addition does not increase cement effectiveness in isotropisation. In addition, it can be seen that the dense samples are closer to the isotropic response, indicating the efficiency of compaction in reducing anisotropy.

**Fig. 3:**
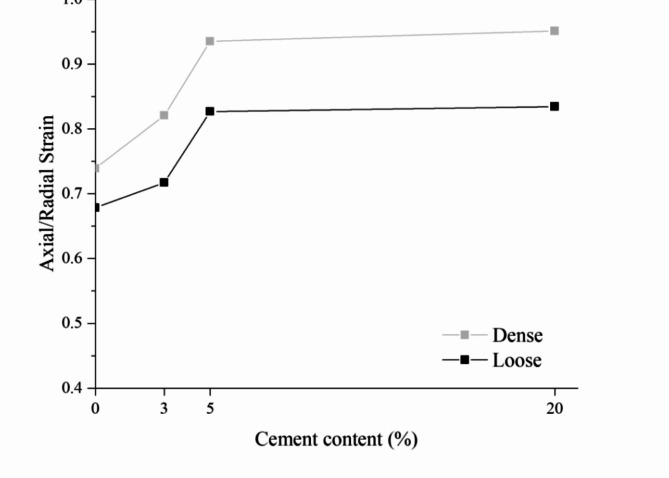
Axial strain/radial strain ratio at 1% axial strain IOT-Portland cement blends.

Figure 3; Table 2 present the axial strain–radial strain ratio for 1.0% radial strain at isotropic compression tests at loose and dense conditions, considering cement contents of zero, 3%, 5%, and 20%. A value of 1% of the radial strain was selected to investigate the behaviour closer to the elastic region while reducing the effects of settling and bedding errors for smaller strain values. It is quite clear from the results that denser conditions reach higher axial strain-radial strain ratios (closer to isotropic behaviour) and that increasing cement content also increases the ratio up to a limiting content of 5% cement for both loose and dense conditions. The most anisotropic condition is for the uncemented specimen at loose condition, in which the axial strain-radial strain ratio for 1.0% radial strain is 0.68 (while the ratio for the uncemented specimen at dense condition is 0.74). In the opposite sense, the nearly isotropic situation (ratio equal to 1) is observed for 20% of cement in the dense condition, with a ratio of 0.95. From Fig. 3, it is possible to observe that from uncemented (zero cement) to slightly (3% cement) and moderately cemented (5%) conditions, the ratio increases almost linearly. In comparison, from moderately cemented (5%) to highly cemented (20%) conditions, the ratio does not change: 0.83 represents loose specimens, while 0.93–0.95 characterises dense specimens. This indicates a limiting cement content that is beneficial to isotropisation. If more cement is added (5% up to 20%), there will possibly be no increase in isotropisation; that is, more cement cannot erase fabric differences in different loading directions generated during compaction. Increasing cement content can reduce the number of large pores and form stronger bridges, preventing particle rotation and, consequently, rearrangement^40–42^.

In the mining context, some considerations can be drawn for the results in Table 2; Fig. 3. Anisotropy intricates modelling by adding material parameters to be determined in the different loading directions. Thus, isotropisation can simplify the problem and increase safety: an effort occurring in an unpredicted direction would not cause any harm since the properties are similar in all directions. Moreover, compacting tailings is an efficient way to increase isotropisation and reduce the necessary cement amount.

**Table 2:** Axial strain–radial strain ratio for 1.0% radial strain at isotropic compression tests.

Test	Cement content—C (%)	Dry unit weight—γ_d_ (kN/m³)	Radial strain	Axial strain	Axial/radial strain ratio
L0C	0	17	1.00	0.68	0.68
L3C	3	17	1.00	0.72	0.72
L5C	5	17	1.00	0.83	0.83
L20C	20	17	1.00	0.83	0.83
D0C	0	19	1.00	0.74	0.74
D3C	3	19	1.00	0.82	0.82
D5C	5	19	1.00	0.93	0.93
D20C	20	19	1.00	0.95	0.95

Anisotropy in granular materials arises at least from three sources: irregular distribution of particle contacts, preferential orientation of voids, and preferential orientation of non-spherical particles^43^. For cylindrical moist tamped specimens, as in this research, cross-anisotropic behaviour would be expected due to a preferred particle orientation in the direction of the compaction effort^44,45^. The angular nature of tailings particles can amplify this anisotropic response^46^. On the other hand, adding cement to tailings reduced the anisotropic behaviour through void-filling, particle coating, and changing contact behaviour^26,42,47,48^. Bonding between particles can modify the force chains, reducing the dispersity in the orientation of contact normals.

**Fig. 4:**
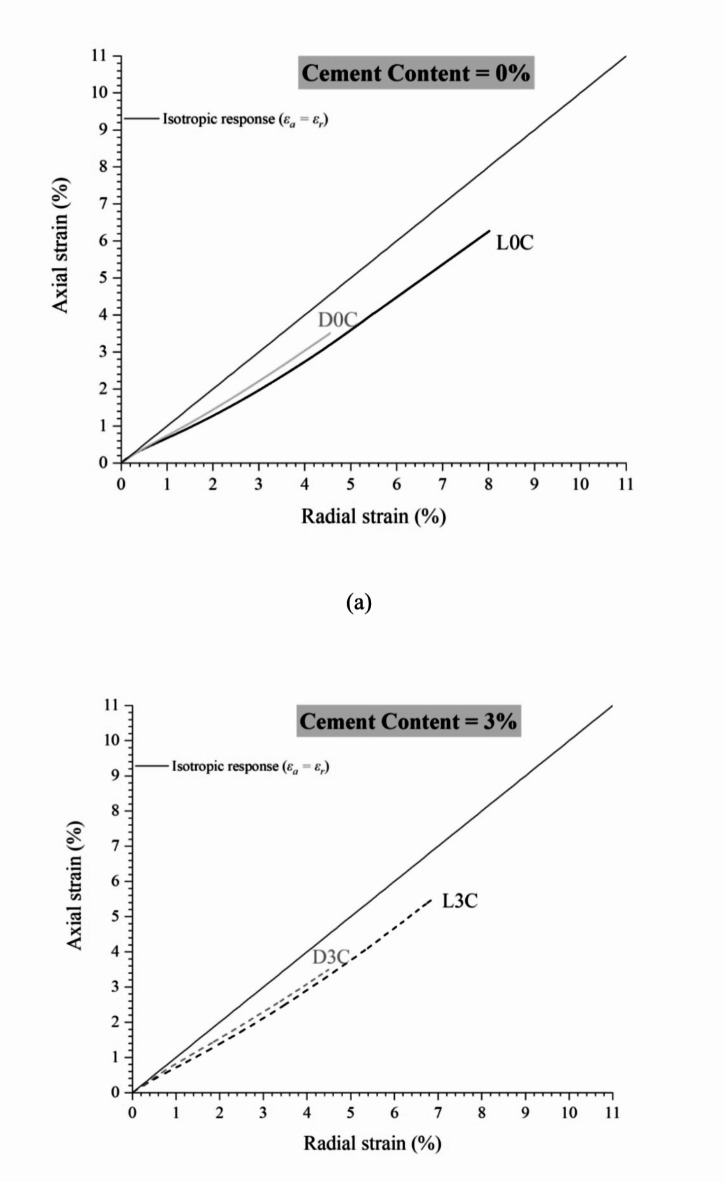

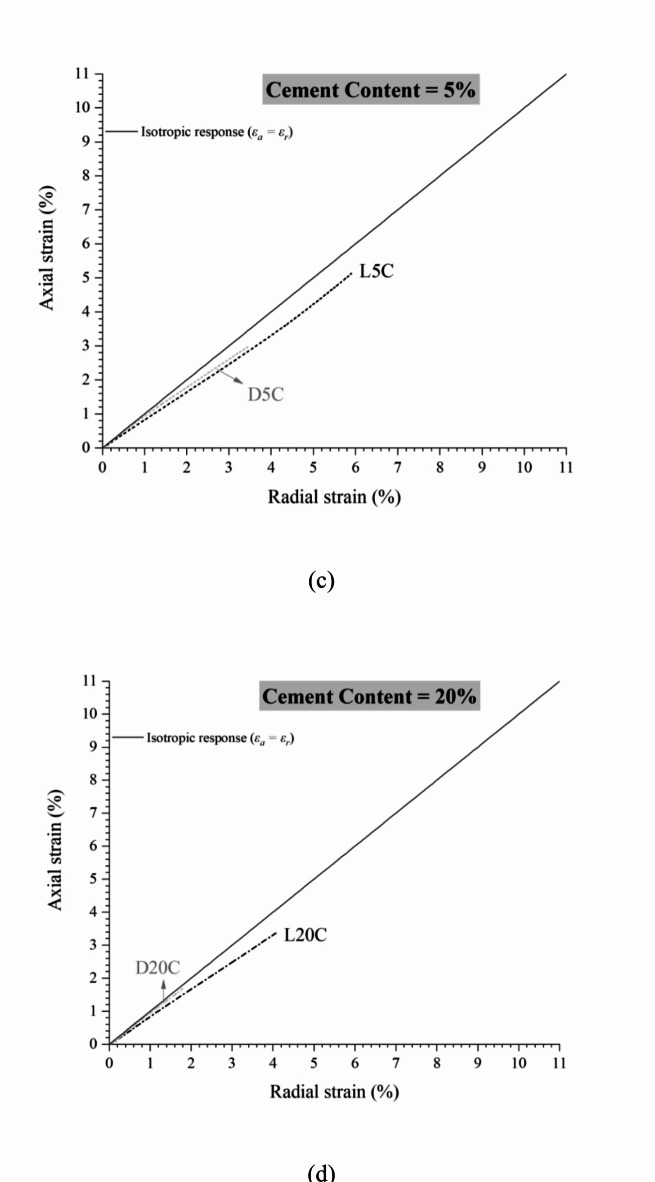
Effect of moulding dry unit weight and cementation on isotropic compression behaviour of IOT-Portland cement mixtures: (a) zero, (b) 3%, (c) 5%, and (d) 20% cement content.

The influence of compaction conditions in the strains’ magnitude and isotropisation can be seen in Fig. 4–d (considering zero to 20% cement content, respectively). From Fig. 4, the higher compaction degree (denser specimens) results in greater isotropisation. The higher the densification, the lower the anisotropy effects caused by axial efforts from specimen moulding (as those applied in the field during compaction). In the denser state, the particles are closer together, facilitating the formation of bonds between them. This cementation usually occurs around the particles, regardless of direction, resulting in a similar axial and radial response and reducing the effects of inherent anisotropy caused by the sample preparation method^26^. Also, it is noted that the magnitude of total axial and radial strains at the end of compression reduces with increasing cement content and compaction degree. For example, the uncemented tests (Fig. 4a) achieve about 3.5% and 6.3% of axial strains (for dense and loose conditions, respectively) for the confining pressure of 120 MPa. On the other hand, the highly cemented (20%) tests (Fig. 4d) achieve axial strains of about 1.7% and 3.4% (for dense and loose conditions, respectively) for the same stress level (120 MPa). Other authors^26,49^ have obtained similar responses with isotropisation increasing for higher cement contents.

### Yielding and convergence

**Fig. 5:**
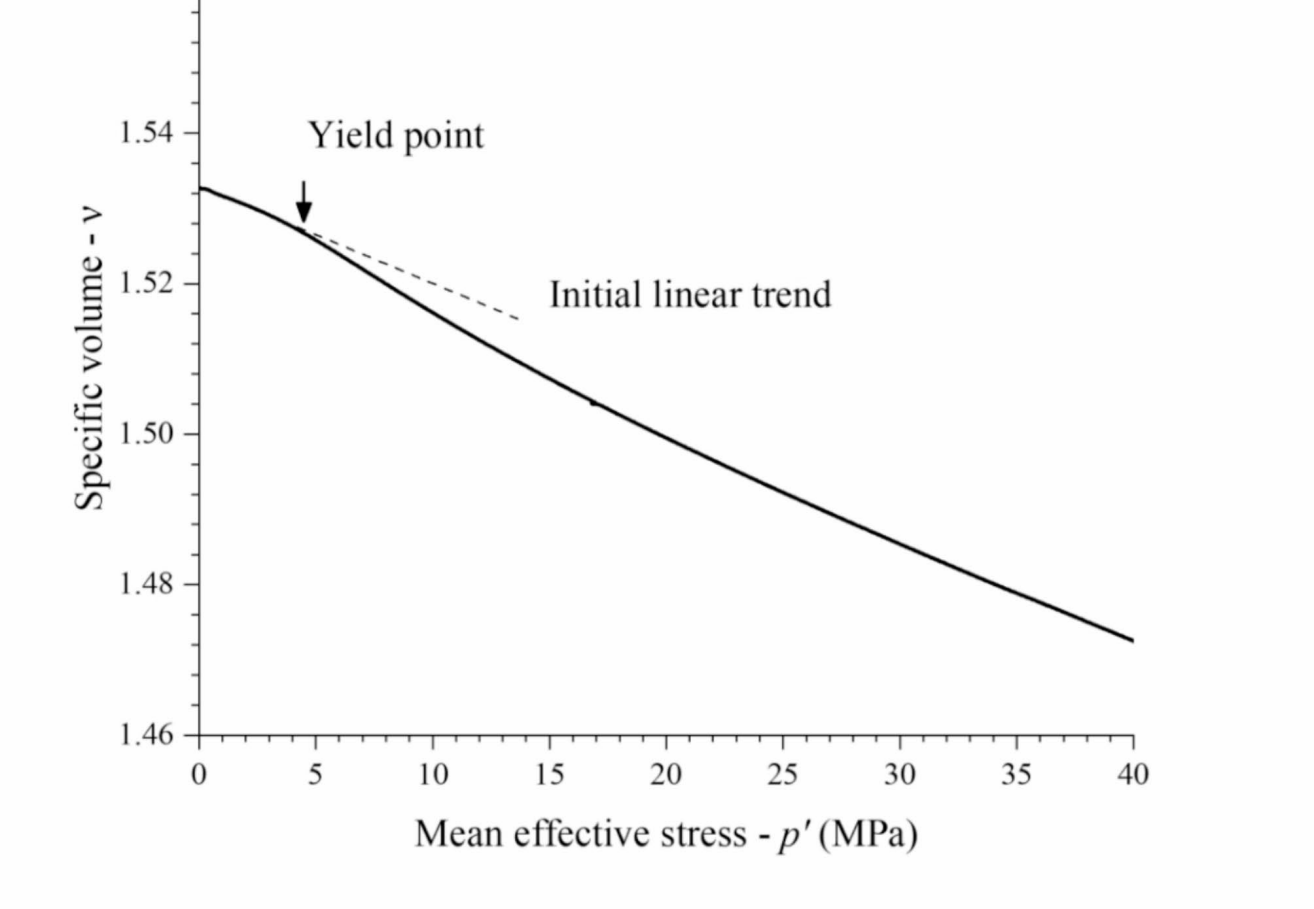
Determination of primary yield points for IOT-Portland cement mixtures according to Rotta et al.^50^.

**Fig. 6:**
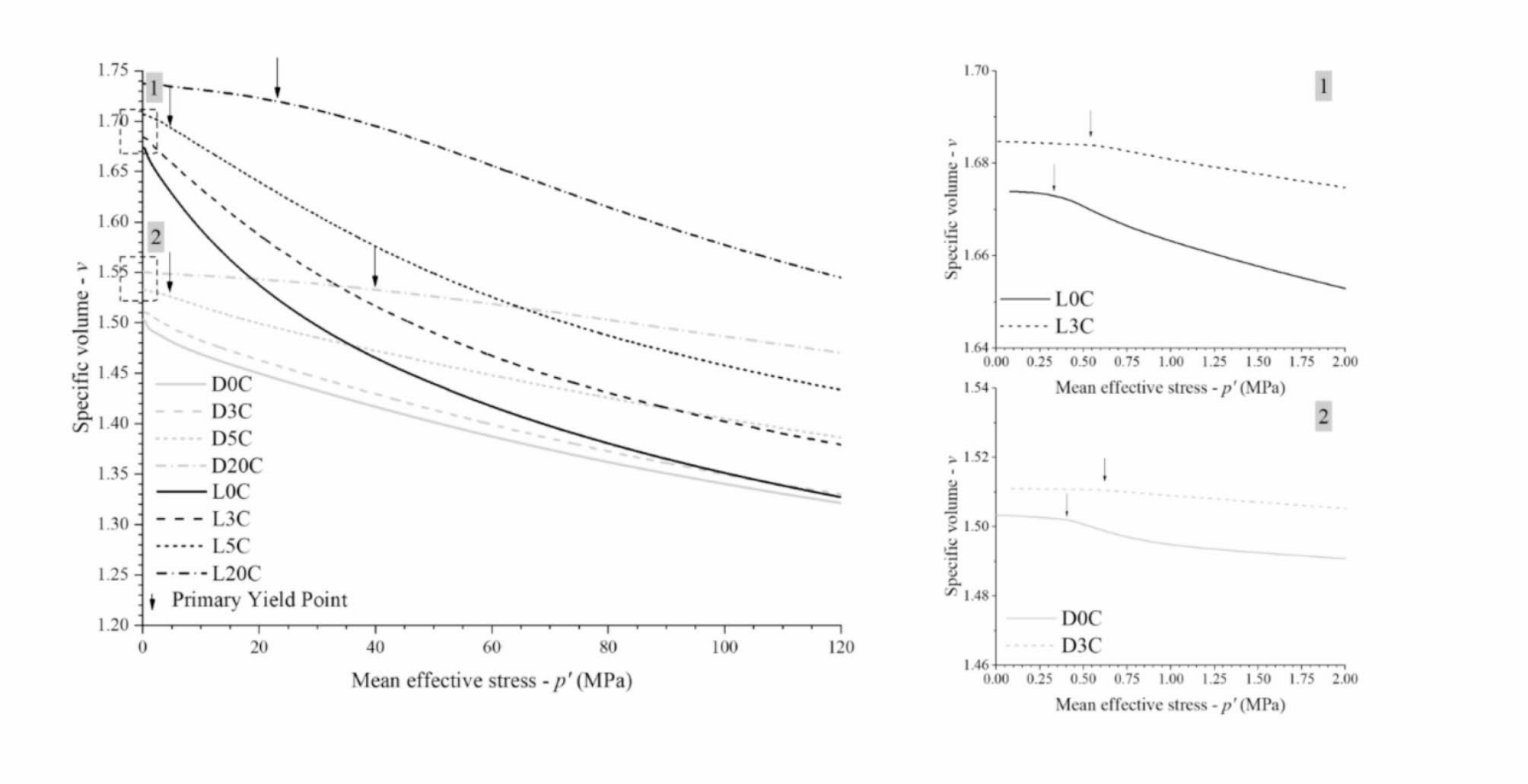
Isotropic compression response for zero, 3%, 5% and 20% cement content and determination of primary yield stress in isotropic compression.

The determination of primary yield points for IOT-Portland cement mixtures was carried out according to Rotta et al.^50^, as exemplified in Fig. 5. The primary yield occurs when *n–p’* relationship loses linear condition. Figure 6 presents the results of the primary yield point under isotropic compression for the eight tests carried out on loose and dense specimens considering cement contents varying from zero to 20%. Table 1 presents the specific isotropic compression primary yield pressures. For the uncemented specimens, loose to dense compaction conditions increased the primary yield stress from 300 to 400 kPa. For the specimens with 3% cement content, the primary yield stress increased to 590 kPa and 605 kPa, respectively, from loose to dense specimens, meaning a yield pressure enhancement of about 50–100% related to uncemented specimens. Increasing cement content from 3 to 5% caused an increase of about 620% for both loose and dense specimens. Finally, increasing cement content from 5 to 20% caused an increase in yield pressure of about 440–800% for loose and dense specimens, respectively.

**Fig. 7:**
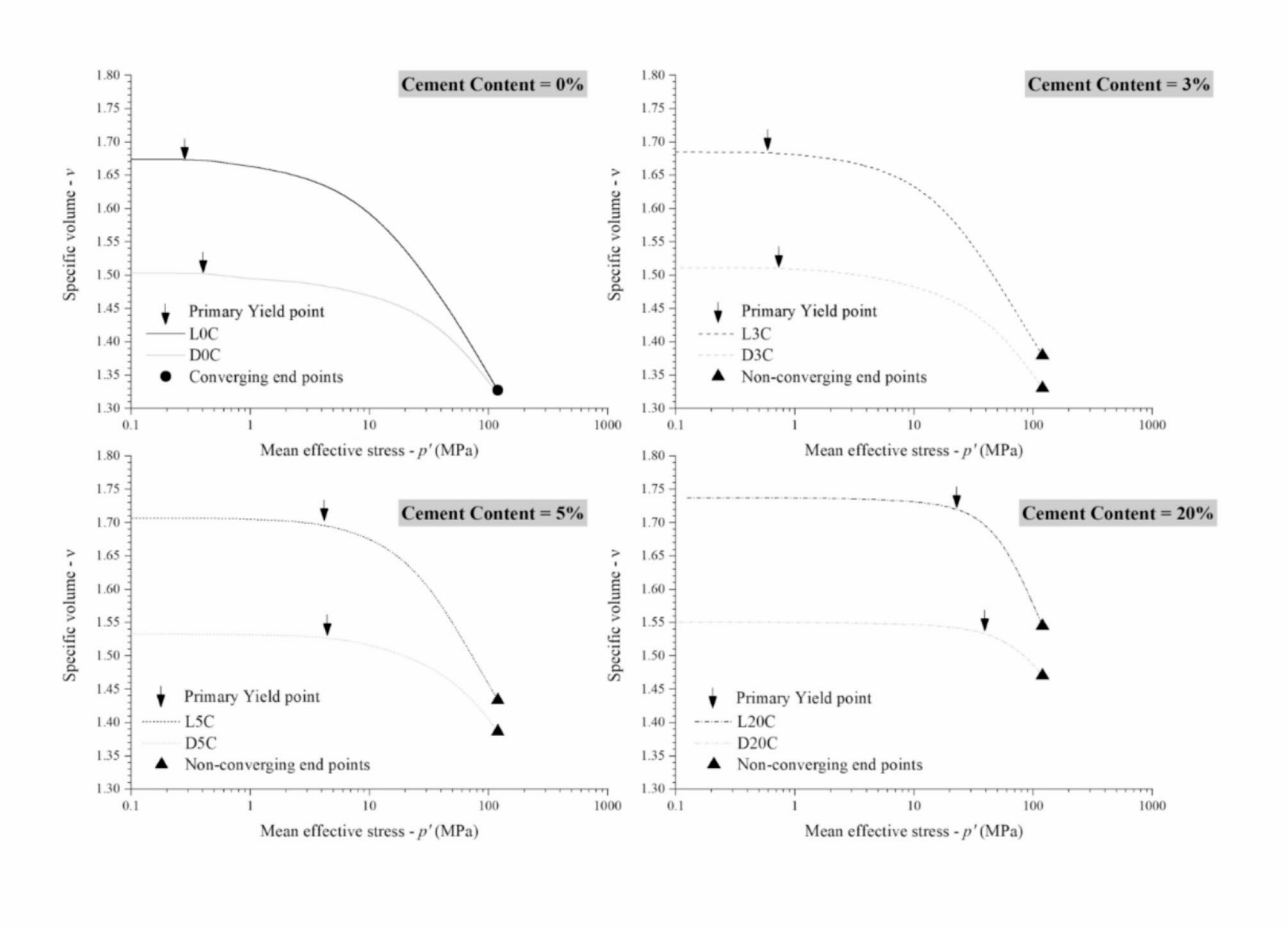
Isotropic compression *n*–log *p’* results for IOT—Portland cement (zero, 3%, 5% and 20%) blends for pressures up to 120 MPa.

Figure 7 compares the results in the *v−*ln *p’* plane for each dosage. The yield points previously defined are highlighted in the curves of each test. In this semi-log space, the higher the cement content, the longer the stiff linear response up to the primary yield point. The compression paths for uncemented tailings (Fig. 7a) are nonlinear almost from the beginning of isotropic loading, while those results for the 20% cement blend (Fig. 7d) are linear up to stresses of approximately 20 MPa for the looser specimen and 40 MPa for the denser specimen. Also, in Fig. 7, after the primary yield point, the specimens follow the compression path up to a linear portion of the curve, named the Post Yield Compression Line (PYCL)^11,50^. Structure allows the material to exist at higher void ratios than non-microstructured materials. Thus, the states achievable in a stress-density space are enlarged when the cement amount increases, represented by the expansion of the PYCL in the *ν*–ln *p’* plane^10^. It can also be observed in Fig. 7 that both loose and dense specimens converged to a unique PYCL at high isotropic stresses (within the stress spectrum studied) only for uncemented tailings. In contrast, the PYCLs for the cemented tailings were considered as those of the loose specimens because the dense ones do not converge up to 120 MPa.

In natural sands, the convergence towards a unique PYCL is usually attributed to the occurrence of particle breakage; also, the slope and location of the PYCL may be directly linked to the particle strength^51–53^. In the case of tailings, Fig. 1 demonstrates the small breakage occurring in the uncemented material compressed up to 120 MPa. Thus, the slow convergence of tailings can be related to distinct breakage patterns in tailings due to their particles’ characteristics^19,54^. The cement can potentialize these effects, causing the non-convergence. Cementation between particles may create persistent elements of fabric and reduce particle rearrangement inside the granular matrix, preventing breakage and causing late convergence. Furthermore, cement lumps and particle aggregates inside the granular matrix can change the force chains and continue to influence the behaviour up to high pressures^55^.

**Fig. 8:**
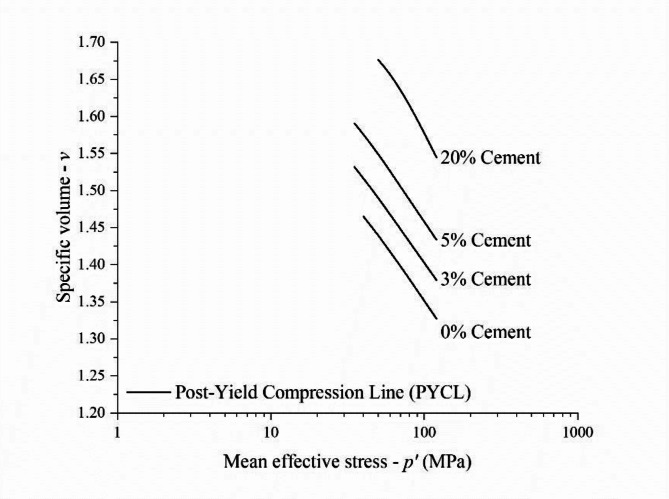
Post Yield Compression Lines (PYCL) for IOT—Portland cement (zero, 3%, 5% and 20% cement content) blends for pressure up to 120 MPa.

Figure 8 presents the combination of PYCL for all the cement contents studied. As verified by Consoli and Foppa^10^, a unique PYCL was obtained for each cement dosage. This indicates that cementation would still have an effect under extremely high pressures, translating the achievable states’ space boundary. This behaviour feature is expected for geomaterials with different degrees of cementation and was first reported by Coop and Atkinson^11^ for carbonate sands.

The slowly convergent or non-convergent isotropic compression paths found for the specimens tested with different initial compaction and cementation conditions represent an attention point during dry stacking design and field execution, especially to the range of pressures that will really occur in mining facilities. The concept observed in Fig. 7 is that the denser the compaction of the iron ore tailings, the larger the mean effective stress during triaxial shearing that will cause volumetric dilation during drained shearing or generate negative pore pressure during undrained shearing, which increases the structure safety by reducing the possibility of static liquefaction^19^. Hence, the stacking raising strongly depends on the initial states achieved by the stack compounding geomaterial through compaction. Moreover, adding cement has potentialized these effects, causing an even slower convergency rate (Fig. 7). This reinforces the need for rigorous compaction control at the densest possible conditions in the field.

### Porosity/cement content index

The unconfined compressive strength (*q*_*u*_) and stiffness of mixtures were analysed under the porosity/cement content index (*η/C*_*iv*_) framework^8,25,56^. According to Consoli et al.^25^, the *η/C*_*iv*_ accounts for changes in compaction and cementation and provides a rational method for the dosage of cement blends controlling aspects such as strength and stiffness^34,57^ and durability^58,59^. In other words, specimens with the same *η/C*_*iv*_ are expected to respond similarly. This means that, for practical reasons, the amount of cement and the degree of compaction can be determined for a given target strength and stiffness required in design. In the context of tailings management, the *η/C*_*iv*_ framework provides means to technological control in the field since the target density and cement can be verified and, if needed, a simple procedure such as the UCS test can be used to validate the dosage methodology.

**Fig. 9:**
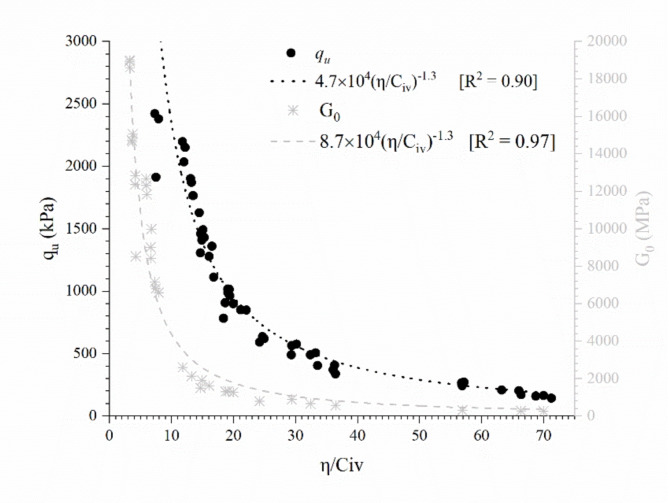
Unconfined compressive strength (*q*_*u*_) and initial shear modulus (*G*_*0*_) versus porosity/cement content index.

Under these considerations, Fig. 9 allows assessing the strength and stiffness of the tailings-cement mixtures. For both *q*_*u*_ and *G*_*0*_, a curve in the form of Eq. (2) was adjusted.

2 $${q_u}~or~{G_0}=A{\left( {{\raise0.7ex\hbox{$\eta $} \!\mathord{\left/ {\vphantom {\eta {{C_{iv}}}}}\right.\kern-0pt}\!\lower0.7ex\hbox{${{C_{iv}}}$}}} \right)^{ - c}}$$

where *A* is a scalar, and *c* is an external adjustment exponent related to the relative influence of cementation and compaction. According to the theoretical derivation of Diambra et al.^56^ and experimental results from many distinct cemented sands^25^, *c* is 1.3 for sandy materials, and *A* is fundamentally associated with the strength of the mineral and cemented phases. Consoli et al.^8^ confirmed the value of *c* = 1.3 for silty sand IOT, the same value as the one used in this study. Equations (3) and (4) represent the best-fit curves relating *h/C*_*iv*_ with *q*_*u*_ and *G*_*0*_, respectively.

3 $${q_u}\left( {kPa} \right)=4.7 \times {\left( {{\raise0.7ex\hbox{$\eta $} \!\mathord{\left/ {\vphantom {\eta {{C_{iv}}}}}\right.\kern-0pt}\!\lower0.7ex\hbox{${{C_{iv}}}$}}} \right)^{ - 1.3}}$$

4 $${G_0}\left( {MPa} \right)=8.7 \times {\left( {{\raise0.7ex\hbox{$\eta $} \!\mathord{\left/ {\vphantom {\eta {{C_{iv}}}}}\right.\kern-0pt}\!\lower0.7ex\hbox{${{C_{iv}}}$}}} \right)^{ - 1.3}}$$

**Fig. 10:**
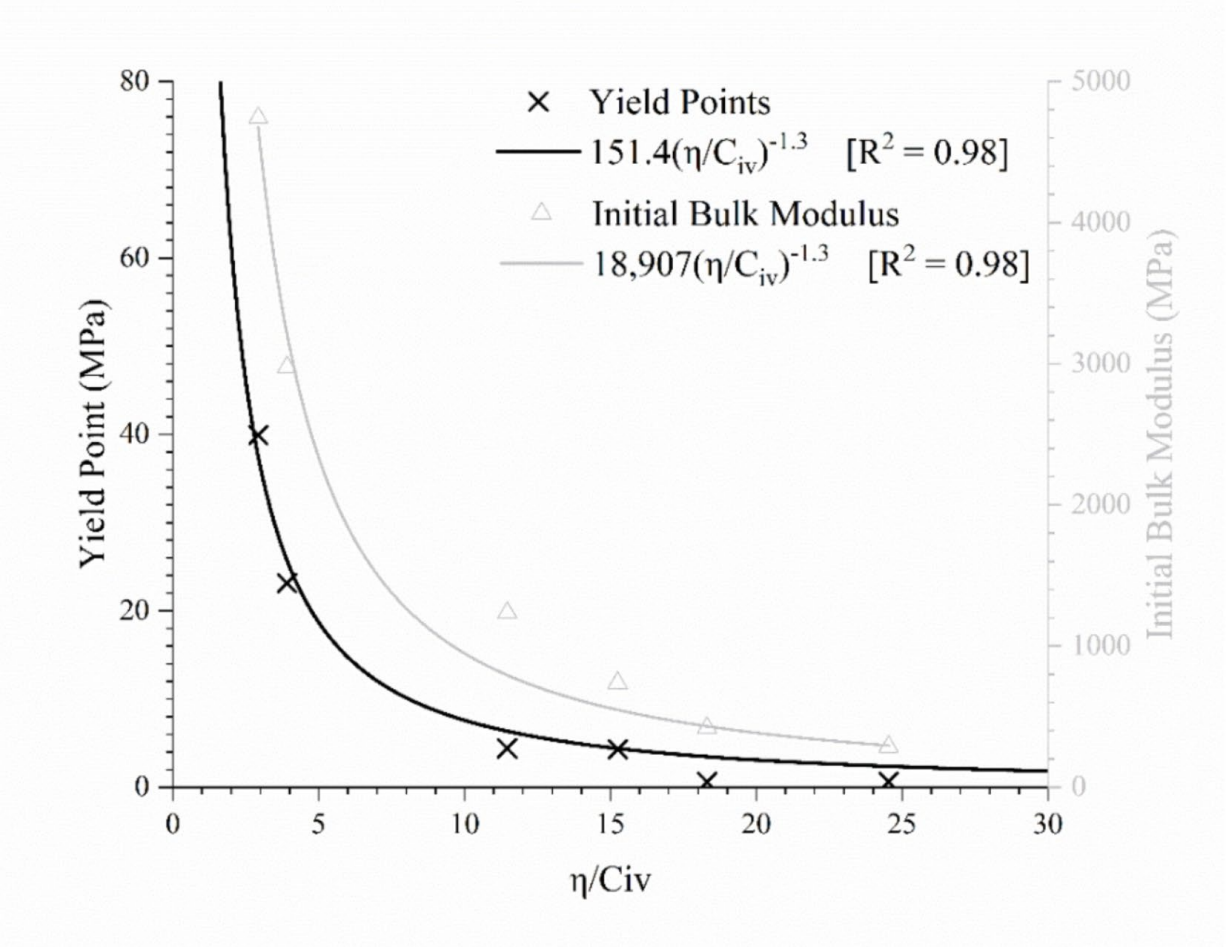
Initial bulk modulus (*B*) and primary yield point (*YP*) under isotropic compression versus porosity/cement content index.

Furthermore, two parameters obtained from the isotropic compression tests (the initial bulk modulus—*B* and the primary yield point—*YP*) were also related to the porosity/cement content index. The results are presented in Fig. 10. The reasonable adjustment of these parameters indicates that the *η/C*_*iv*_ index controls the behaviour of IOT-Portland cement mixtures up to isotropic stresses equivalent to the primary yield point. Equations (5) and (6) represent the best-fit curves relating *h/C*_*iv*_ with *B* and *YP*, respectively.

5 $$B\left( {MPa} \right)=18,907 \times {\left( {{\raise0.7ex\hbox{$\eta $} \!\mathord{\left/ {\vphantom {\eta {{C_{iv}}}}}\right.\kern-0pt}\!\lower0.7ex\hbox{${{C_{iv}}}$}}} \right)^{ - 1.3}}$$

6 $$YP\left( {MPa} \right)=151.4 \times {\left( {{\raise0.7ex\hbox{$\eta $} \!\mathord{\left/ {\vphantom {\eta {{C_{iv}}}}}\right.\kern-0pt}\!\lower0.7ex\hbox{${{C_{iv}}}$}}} \right)^{ - 1.3}}$$

**Fig. 11:**
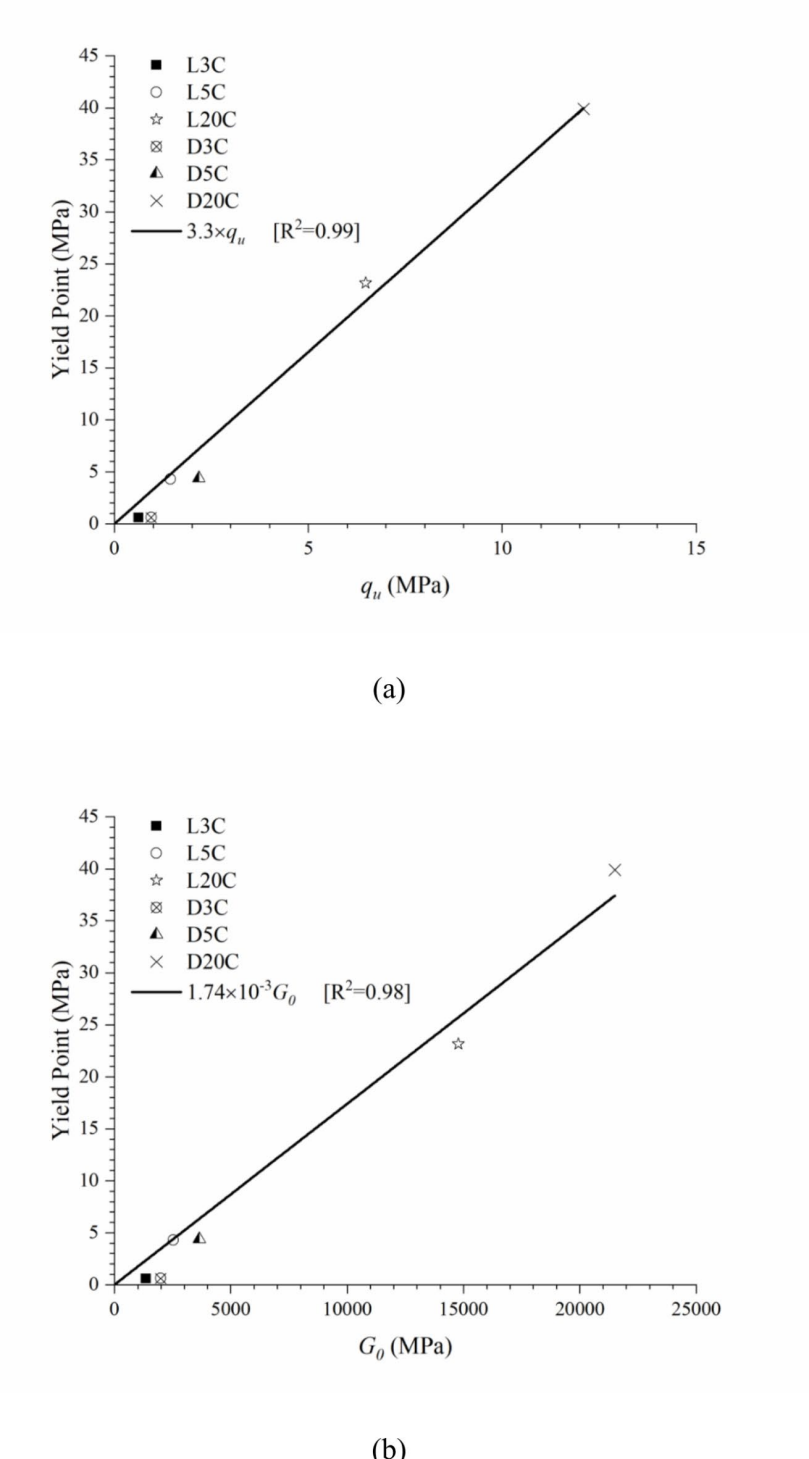
Relationship between (a) unconfined compressive strength (*q*_*u*_) and primary yield point (*YP*) under isotropic compression, and (b) initial shear modulus (*G*_*0*_) and primary yield point (*YP*) under isotropic compression.

Then, it is verified that the *η/C*_*iv*_ index controls important plastic (*q*_*u*_ and *YP*) and elastic (*G*_*0*_ and *B*) parameters, which can be useful in the design of compacted filtered tailings. However, as demonstrated in Fig. 8, the cement content mostly influences the post-yield behaviour. Thus, the porosity/cement content could represent well the behaviour up to the primary yield point. After this point, the primary deformation mechanism is no longer related to the cemented structure because of the degradation of the bonded structure. The behaviour is then influenced by the change in the material gradation (and its physical characteristics) caused by the inclusion of cement particles. Furthermore, the fit equations obtained have the same form for all parameters, only changing the scalar value (*A*). Thus, beyond relating the design parameters to the *η/C*_*iv*_ index, it is possible to obtain direct relationships amongst them. Figure 11a and b show the correlation between the primary yield point (*YP*) with *q*_*u*_ (see Eq. (7)) and *G*_*0*_ (see Eq. (8)), respectively. As expected, a linear trend is observed for both relationships.

7 $$YP\left( {MPa} \right)=3.3 \times {q_u}\left( {MPa} \right)$$

8 $$YP\left( {MPa} \right)=0.00174 \times {G_0}~\left( {MPa} \right)$$

Equations (7) and (8) are essential correlations that allow empirically connecting two parameters (*q*_*u*_ and *G*_*0*_) that can be measured in basic laboratories with a parameter (YP) that requires special equipment to be measured. Of course, the above equations are restricted to the presently studied geomaterials, and further studies must be carried out for other tailings and cements to check possible generalisations. Although *G*_*0*_ is an elastic parameter and *YP* is a plastic one, it is expected that yield could be related to a certain modulus degradation degree. For cemented materials, this degradation is negligible until bonds break; then, only minor deviations from the initial shear modulus are expected.

## Concluding remarks

The behaviour of IOT—Portland cement mixtures considering high pressure (up to 120 MPa) isotropic compression, unconfined compression and ultrasonic pulse velocity tests was carried out in the present research. The results assessed the contribution of cement content and compaction conditions to isotropisation, compressibility (bulk modulus—*B*), changes in the isotropic primary yield point (*YP*), post-yield compression line (PYCL) and the variation in *q*_*u*_ and *G*_*0*_ as a function of porosity and cement content. The main conclusions of the study are presented below:

- Increasing cementation and compaction can reduce the degree of anisotropy and magnitude of volumetric strains of iron ore tailings during isotropic compression. Cement bridges reduce inherent anisotropy from the preferred orientation of particles and voids. The isotropisation enhances the predictability of tailings behaviour using existing methods, as modelling anisotropic materials remains challenging.
- The addition of cement expands the PYCL in the *ν*–ln *p’* plane, amplifying the geomaterial’s range of achievable states. This suggests that cementation would continue to have a significant impact even under extremely high pressures. Also, cement has prevented convergence towards unique PYCL. Therefore, rigorous compaction control is required in the field since the initial condition of cemented tailings determines their behaviour up to high pressures.
- The porosity/cement content index (*η/C*_*iv*_) controls the strength (*q*_*u*_) and the stiffness parameters (initial shear modulus—*G*_*0*_, initial bulk modulus—*B*) of IOT-Portland cement mixtures. The initial bulk modulus is only predicted by *η/C*_*iv*_ up to stresses equivalent to the primary yield point because, after this, the primary deformation mechanism is no longer related to the cemented structure due to bonding degradation. Thus, the index proves efficient for field control and dosage of iron ore tailings-Portland cement mixtures for application in dry stacks. Both moduli (*G*_*0*_ and *B*) are crucial parameters in structure design because they control the deformations expected to occur during safe operational conditions long before the failure.

In summary, the initial *η/C*_*iv*_ could satisfactorily represent the mixtures’ behaviour up to the primary yield point (Fig. 10), which can be sufficient for a wide range of applications. Thus, it is possible to conclude that the *η/C*_*iv*_ index is an efficient parameter for determining the mechanical response of iron ore tailings-Portland cement mixtures while the cementation is still acting and influencing the behaviour. When the stress (or strain) level in which cementation breaks is achieved, the cementation is no longer the primary deformation mechanism, and the initial *η/C*_*iv*_ no longer defines the behaviour. The results indicate that the porosity/cement content framework can be efficiently used for control and design aspects, providing an efficient index in determining cement tailings behaviour, which can be obtained from simple tests.

## Data Availability

The data supporting this study’s findings are available from the corresponding author upon reasonable request.

## List of symbols

B_r_: Relative breakage parameter
B: Initial bulk modulus
C_c_: Coefficient of curvature
C_u_: Coefficient of uniformity
C_iv_: Volumetric cement content
G_S_: Specific gravity
G_0_: Initial shear modulus
IOT: Iron ore tailings
p’: Mean effective stress
PYCL: Post-yield compression line
YP: Primary yield point
e_a_: Axial strain
e_r_: Radial strain
e_v_: Volumetric strain
g_d_: Dry unit weight
g_s_: Unit weight of solids
ν: Specific volume = 1 + e
h: Porosity
q_u_: Unconfined compressive strength
